# Comprehensive structural assignment of glycosaminoglycan oligo- and polysaccharides by protein nanopore

**DOI:** 10.1038/s41467-022-32800-4

**Published:** 2022-08-30

**Authors:** Parisa Bayat, Charlotte Rambaud, Bernard Priem, Matthieu Bourderioux, Mélanie Bilong, Salomé Poyer, Manuela Pastoriza-Gallego, Abdelghani Oukhaled, Jérôme Mathé, Régis Daniel

**Affiliations:** 1 Université Paris-Saclay, Univ Evry, CNRS, LAMBE, Evry-Courcouronnes, France; 2 CNRS, CERMAV, University Grenoble Alpes, Grenoble, France; 3 CY Cergy Paris Université, CNRS, LAMBE, Cergy-Pontoise, France

**Keywords:** Single-molecule biophysics, Bioanalytical chemistry

## Abstract

Glycosaminoglycans are highly anionic functional polysaccharides with information content in their structure that plays a major role in the communication between the cell and the extracellular environment. The study presented here reports the label-free detection and analysis of glycosaminoglycan molecules at the single molecule level using sensing by biological nanopore, thus addressing the need to decipher structural information in oligo- and polysaccharide sequences, which remains a major challenge for glycoscience. We demonstrate that a wild-type aerolysin nanopore can detect and characterize glycosaminoglycan oligosaccharides with various sulfate patterns, osidic bonds and epimers of uronic acid residues. Size discrimination of tetra- to icosasaccharides from heparin, chondroitin sulfate and dermatan sulfate was investigated and we show that different contents and distributions of sulfate groups can be detected. Remarkably, differences in α/β anomerization and 1,4/1,3 osidic linkages can also be detected in heparosan and hyaluronic acid, as well as the subtle difference between the glucuronic/iduronic epimers in chondroitin and dermatan sulfate. Although, at this stage, discrimination of each of the constituent units of GAGs is not yet achieved at the single-molecule level, the resolution reached in this study is an essential step toward this ultimate goal.

## Abstract (web summary)

Glycosaminoglycans (GAGs) are highly anionic functional polysaccharides with subtle structural variations that are very difficult to differentiate. Here the authors demonstrate proof-of-concept single-molecule detection by nanopore, taking a first step towards the ultimate goal of GAG sequencing.

## Introduction

**Fig. 1:**
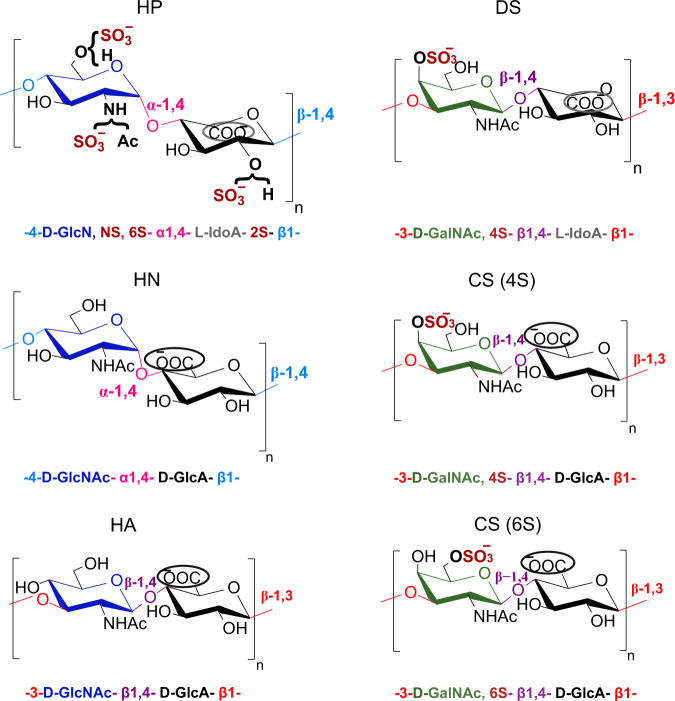
Constituent disaccharide units of the different GAGs studied: heparin (HP), heparosan (HN), hyaluronic acid (HA), dermatan sulfate (DS), and chondroitin sulfate (CS). The structural determinants studied are represented by different colors for the different types of monosaccharide units (d-Glc (dark blue), d-Gal (green), or uronic acid (black)), the type of glycosidic linkage within each disaccharide repeating unit (α−1,4 or β−1,4) and also between the disaccharide units (β−1,4 or β−1,3), the epimer position of the carboxylate group in uronic acid (encircled carboxylic acid at up (d-GlcA) or down (l-IdoA)) and also the presence or absence of sulfate groups.

Glycosaminoglycans (GAGs) are highly anionic linear polysaccharides expressed on the cell surface and in the extracellular matrix, which have prominent roles in a variety of physiological and pathological processes, through the binding of numerous proteins (e.g. chemokines, growth factors)^1,2^. Given the central biological functions of GAGs and considering them as promising pharmacological targets, there is a strong demand for methods capable of reading the GAGs oligosaccharide sequences, especially those that are specifically involved in pathological situations. While such tools are widely available for other classes of biopolymers (DNA, proteins), efficient sequencing methods for GAGs based on routine analytical tools are still lacking. This is due to the remarkable molecular complexity of GAGs. Indeed, a puzzling feature of GAGs such as heparan sulfate (HS) and chondroitin sulfate (CS) is that they are regioselectively modified at the polysaccharide level through epimerization, *N*- and *O*-sulfation, and deacetylation, thus leading to complex patterns that are finely tuned for specific interactions^3^. These two emblematic groups of GAGs HS/Heparin (HP) and CS/dermatan sulfate (DS), are composed of alternating residues of hexosamine and uronic acid (UA) forming a disaccharide repeating building block, linked α-(1,4) in HS/HP and β-(1,3) in CS (Fig. 1). Hexosamine motif is an *N*-acetyl-d-galactosamine (GalNAc) in CS, and *N*-acetyl-d-glucosamine (GlcNAc) in HS/HP in which the *N*-acetyl group could be further deacetylated and sulfated (GlcNS) (Fig. 1). Uronic acid is present under two epimer forms, namely d-glucuronic (GlcA) and l-iduronic acid (IdoA), both of which are found in HS/HP whereas CS contains only GlcA. In addition to these isomeric differences, HS/HP and CS exhibit a considerable variability of sulfation: in addition to sulfation of the amine group in HS/HP, hydroxyl groups can also be sulfated at the position C2 of uronic acid and the position C3 and/or C6 of GlcN and C4 and/or C6 of GalN.

However, the resulting huge combinations of regioselective modifications within disaccharide (degree of monosaccharide polymerization of two, dp2) elementary units lead to an extraordinary structural complexity that conventional bio-structural methods are facing. Despite great progress in structural characterization of GAGs over the past two decades, particularly by mass spectrometry^4^, the need for GAG sequencing methods is still not fulfilled^5,6^. In this context, the recent development of nanopore-based methods to decipher the information encoded in linear biopolymers such as DNA^7^ and proteins^8,9^ could also bring a decisive breakthrough in glycosciences^10,11^. Nanopore analysis remains comparatively much less applied to carbohydrates, especially bioactive polysaccharides such as GAGs. Few studies have been reported on nanopore detection of GAGs, using either solid-state nanopore fabricated from conventional nanofabrication materials such as silicon nitride (SiNx)^12^ or nanoscopic protein channels. Large solid-state nanopores can easily accommodate polysaccharides, and have recently been exploited for the detection of the unsulfated GAG hyaluronan^13,14^, sulfated GAGs heparin and oversulfated chondroitin sulfate contaminant in a pharmaceutical anticoagulant preparation^15,16^, and of heparan sulfate^17^. Nanoscopic solid-state nanopores and biological nanopores can achieve high molecular resolution given their dimension of the same scale as many single molecules^18,19^. Moreover, given the importance of pore length and internal structure in discrimination power, their ability to be tailored with angstrom precision through surface functionalization^20,21^ or protein engineering techniques^22^, respectively, makes them attractive high-resolution sensors. To date, only a few reports have described the use of protein nanopores for the detection of GAGs with oligosaccharide-level resolution^23^. We previously reported the sensitive detection of hyaluronan (HA) oligosaccharides by protein nanopores and their ability to discriminate these unsulfated oligosaccharides according to their size^24^, thus fueling the concept of nanopore-based single-molecule mass spectrometry^25,26^. It allowed real-time recording of oligosaccharide formation upon enzyme depolymerization of HA^27^.

**Fig. 2:**
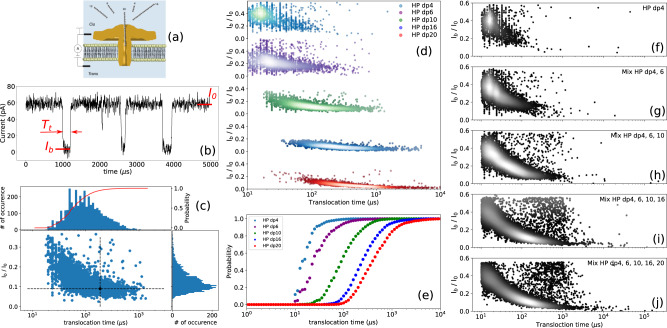
Single-channel detection of heparin (HP) oligosaccharides in the dpn range between tetrasaccharide dp4 and icosasaccharide dp20 (dpn, *n* = 4-20) analyzed individually and in mixture by successive introduction of HP oligosaccharides into the *cis* compartment. **a** Schematics of the experimental setup with aerolysin inserted in a lipid bilayer (not to scale). The ionic current is recorded while applying a constant voltage. **b** Illustration of typical single nanopore current recording in presence of HP dp10. The current is blocked by the presence of the molecule inside the channel (translocation). For each translocation event the blocked current *I*_b_, the open pore current *I*_0_ and the duration of event *T*_t_ are calculated. **c** Scatter plot for HP dp10 with the associated current histogram, translocation time histogram with logarithmic binning and the corresponding integrated probability (red curve and right axis). The black dot represents the first event represented on **b**. The distortion of the event cloud at short timescale is due to the signal distortion from the low-pass filtering of the signal. **d** Scatter plots obtained for different sizes of HP oligosaccharides when they were analyzed individually. Each dot represents the translocation of a single molecule. At least 2000 events were recorded for each molecule type. **e** Integrated probability of translocation for different sizes of HP oligosaccharides. Scatter plots of the **f** HP dp4, **g** mixture of HP dp4 and HP dp6, **h** mixture of HP dp4, HP dp6 and HP dp10, **i** mixture of HP dp4, HP dp6, HP dp10 and HP dp16, and **j** mixture of HP dp4, HP dp6, HP dp10, HP dp16 and HP dp20. The relative density of events on the plots is highlighted by the markers color: the brighter the dot, the denser the events. Progressive appearing of the deeper and longer events is clearly seen by the shift of the clouds of events towards long translocation times. All data were recorded in 4 M LiCl, 25 mM HEPES buffer and 1.0 mM EDTA at pH 7.5, 20.0 °C, at the bias voltage of +60 mV and using 43 μM of each oligosaccharide. For mixture analysis, oligosaccharides were added one by one at the same concentration (10 μM) to the *cis* chamber and the residual current was measured. Source data for this figure are provided as a Source Data file.

In the study herein, we address a much greater structural complexity by targeting the analysis of sulfated oligosaccharides from HS/HP and CS/DS using the aerolysin nanopore (AeL) (Fig. 2a) to probe not only the oligomer length but also fine structural determinants such as stereochemistry of osidic linkage, monomer composition and sulfate distribution along the oligosaccharide chain. Since interactions with partner proteins rely on the specific recognition of these structural determinants, in particular the specific arrangement of sulfate groups in given GAG sequences, the so-called sulfation code, it is of importance to be able to determine them within these sequences, and in this respect the reported nanopore strategy paves the way for this deciphering^28^.

## Results

### Investigating size discrimination

Figure 2a illustrates the organization of the translocation device through the aerolysin nanopore without attempting to respect the scale of the individual players (GAGs and the nanopore). The nanopore assays were carried out in 25 mM HEPES buffer, pH 7.5, containing 1 mM EDTA and 4 M LiCl. Under these experimental conditions and applying a voltage of +60 mV (cathode in the *cis* compartment), an ionic open pore current (*I*_0_) of 58 ± 3 pA was observed (Fig. 2b), allowing the reliable detection of GAG oligosaccharides translocated through the AeL nanopore. More details on the experimental conditions are provided at Supplementary Information (Supplementary Note 1, and Supplementary Figs. 1 and 2). Size discrimination of sulfated GAG oligosaccharides using the AeL nanopore under these experimental conditions was first studied on compounds derived from heparin, a relatively homogeneous GAG because it is almost completely sulfated and consists of repeating disaccharide units [−4-glucosamine NS,6S- α1,4- iduronic acid,2S- β1-]. Introduction of HP oligosaccharides varying in size from dp4 to dp20 into the *cis* compartment generated transient current blockades corresponding to translocation events that were easily distinguishable from noise (Fig. 2b). The choice of even oligosaccharides is determined by the enzymatic mode of production of the oligosaccharides, which exploits the activity of enzymes lyase and hydrolase degrading polysaccharide substrates into even oligosaccharides. The data shows an increase in both the current blockade (Fig. 2c) and the average translocation time (Fig. 2c, e) as a function of the polymerization degree, in agreement with our previous study on non-sulfated HA oligosaccharides^24^, thus showing the capability of AeL nanopore to detect highly sulfated oligosaccharides of different lengths.

Normalized blockade current (ratio of blockade current (*I*_b_) to open pore current (*I*_0_), (*I*_b_/*I*_0_)) decreases from dp4 to dp10, and then barely decreases (about 9 % change in blockade current) above dp10, indicating that the pore is almost completely occupied by dp10 and therefore further increase in oligosaccharide size does not significantly change the depth of the events. For lower oligosaccharide sizes, the relative increase of the blocked current is mostly due to the low pass filter employed in the acquisition setup^29^. The calculated blockade durations for dp4, dp6, dp10, dp16 and dp20 are 19 ± 10 µs, 23 ± 15 µs, 86 ± 10 µs, 230 ± 15 µs and 391 ± 17 µs, respectively. An almost linear increase of the translocation time with the size of the oligosaccharides is observed, despite some deviation from linearity for the smallest oligosaccharides HP dp4 and dp6. We have previously reported the linear dependence of translocation time on the size of oligosaccharides for the non-sulfated glycosaminoglycan hyaluronic acid^24^. This linear dependency is also the case for the glycosaminoglycan CS (discussed later) for which we also determined the same translocation time/size relationship (dp6 = 24 μs, dp8 = 40 μs, dp10 = 48 μs, dp12 = 63 μs, dp16 = 94 μs and dp20 = 109 μs). The dependence of translocation time on the size of oligosaccharides thus appears to be a general trend for GAGs.

**Fig. 3:**
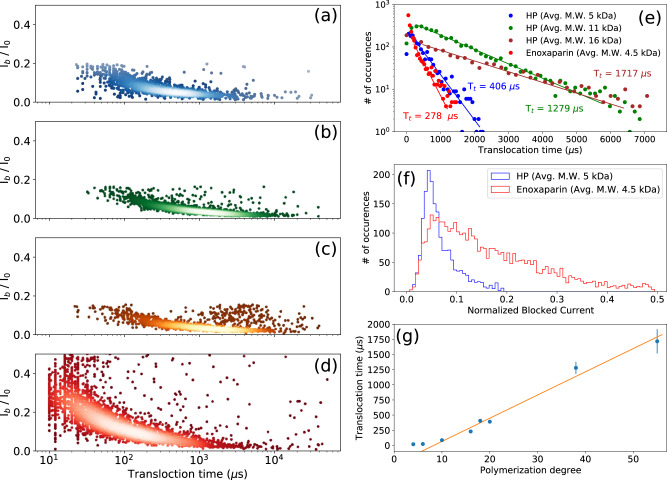
Investigation of size discrimination of heparin polysaccharides using AeL nanopore. Scatter plots derived from HP polysaccharides with the average molecular weights of **a** 5 kDa, **b** 11 kDa and **c** 16 kDa; and **d** of enoxaparin with the average molecular weight of 4.5 kDa. **e** Superimposed translocation time histograms corresponding to **a**–**d** scatter plots. Distinct translocation times are observed for three different sizes of HP polysaccharides. Enoxaparin distribution is clearly not mono-exponential contrarly to other samples. The timescales resulting from mono-exponential fits are 406 ± 15 µs, 1279 ± 30 µs, 1717 ± 73 µs and 278 ± 24 µs for HP 5 kDa (dp18), HP 11 kDa (dp38), HP 16 kDa (dp55) and enoxaparin, respectively. **f** Superimposed *I*_b_ histograms corresponding to **a**, **d** scatter plots. The superimposed *I*_b_ histograms of enoxaparin and 5 kDa HP show that enoxaparin has a rather broad distribution of oligosaccharides compared to 5 kDa HP despite their close average molecular weights. **g** Translocation time versus polymerization degree from data of Figs. 2 and 3e. The straight line is a guide to the eye and the errors bars represent the statistical standard deviation resulting from the curve fitting procedure of the distribution. The mean translocation time would be about 40 µs per monomer. The deviation from the linearity for short oligo length is due to the signal distortion at short timescales. All data were recorded in 4 M LiCl, 25 mM HEPES buffer and 1.0 mM EDTA at pH 7.5, 20.0 °C, and at the bias voltage of +60 mV. Source data for this figure are provided as a Source Data file.

The deviation from the linear dependency of the translocation time on the size of oligosaccharides for the smallest HP oligosaccharides (dp4 and dp6) is due to the cutoff frequency of the low-pass filter (*f*_c_ = 50 kHz) that causes the broadening of the distribution (both in duration and current blockade) of the translocation events at short translocation times and a minimum threshold value of 20 µs (Figs. 2d and 3g)^30,31^. However, this does not preclude the observation of a clear relationship between translocation time and the size of oligosaccharides. This effect, well known in the nanopore field, is well described in the literature data^29^. Shortly, low pass filter of cutoff frequency *f*_*c*_ is equivalent, in the time domain, to a convolution of the signal by a bell shape curve of width 1/ *f*_*c*_ (*i.e*. 20 μs in our experiments). This results in a broadening of the event of 1/ *f*_*c*_. For shorter events, the distortion is thus more pronounced and leads to a shallower blocking of the current and thus a higher mean blocked current.

Nanopore experiments carried out using mixtures of HP oligosaccharides show that our experimental approach can identify the presence of different oligosaccharide species constituting the mixture despite a partial overlap. Scatter plots of the successive introduction of oligosaccharides HP dp4 to dp 20 into the *cis* chamber are shown in Fig. 2f–j. Note that the scatter plot of the mixture cannot be considered as the simple addition of the individual components because this would require considering the entry rate to be equivalent for each component. The scatter plots upon successive additions of longer HP showed the gradual shift of the event population towards the longer translocation times, and the occurrence of deeper events in the current traces. This observation demonstrates the convenience of the AeL nanopore to highlight the polydispersity of the HP oligosaccharides mixtures.

The size discrimination capability of the AeL nanopore was also investigated at the polysaccharide level by analyzing full size heparin (16 kDa, corresponding to dp55), and low molecular weight heparin preparations (11 kDa and 5 kDa, i.e. dp38 and dp18, respectively). As expected from the above results with HP dp16 and dp20, these polysaccharides almost completely occupy the pore, thus leading to similar normalized current blockades for the three heparins (Fig. 3a–c). However, they can be distinguished through distinct translocation times (Fig. 3e). The calculated event duration for the 16 kDa HP (1717 ± 200 µs) is almost four times larger than that of the 5 kDa HP (406 ± 20 µs). This confirms the linear dependence of the translocation time with chain length as shown in Fig. 3g, suggesting that, in principle, there is no size limit for polysaccharide analysis based on the translocation time criterion.

Afterwards, the nanopore analysis technique was applied to enoxaparin (Fig. 3d), a low-molecular weight heparin (4.5 kDa) used in clinical applications as an anticoagulant medication for the treatment of deep vein thrombosis and pulmonary embolism as well as acute coronary syndrome and heart attacks^32^. Given the close average molecular weights of enoxaparin and the aforementioned 5 kDa HP, their translocation behaviors were compared. Both enoxaparin and the 5 kDa preparation are low molecular weight heparins, thus sharing the same structural organization. Their superimposed *I*_b_ histograms (Fig. 3f) reveal that enoxaparin has a fairly wide distribution of oligosaccharide sizes compared to 5 kDa HP in agreement with their size exclusion chromatography results (Supplementary Fig. 3). This result illustrates the sensitivity of the AeL pore to the identity of analyte and its utility for quality control analysis of clinical heparin.

By comparing the scatter plot of enoxaparin with that of the mixture of HP oligosaccharides, it can be qualitatively concluded that enoxaparin contains almost all the oligosaccharides from dp4 to dp20 in agreement with the literature data^33,34^ (Supplementary Fig. 4). Note that because of the wide distribution of the oligosaccharide population, it is difficult to perform a quantitative analysis.

### Degree of sulfation

**Fig. 4:**
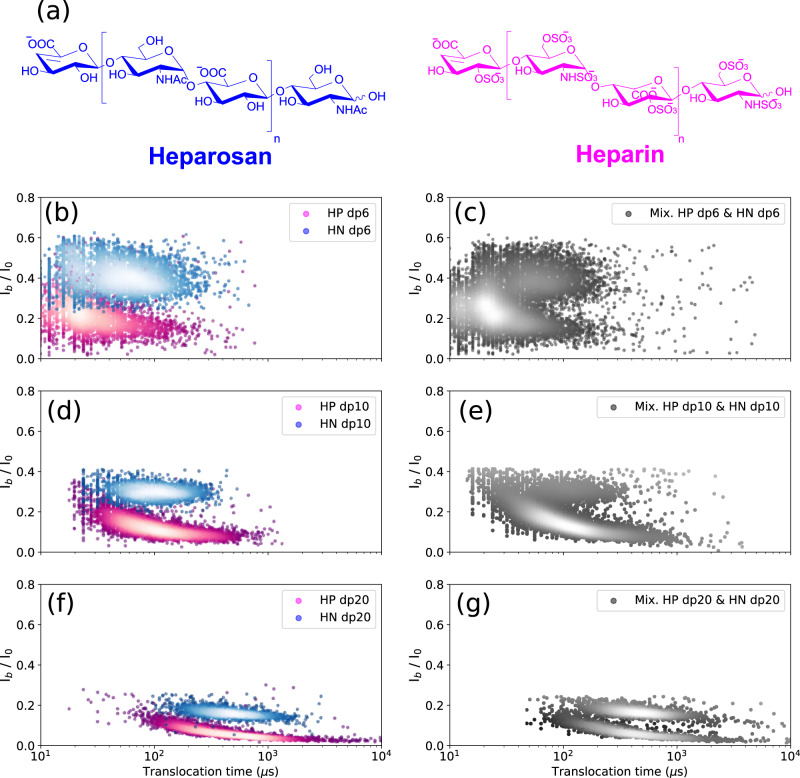
Discrimination of the non-sulfated heparosan and fully sulfated heparin oligosaccharides analyzed individually and in mixture. **a** Structures of the heparosan and heparin. Superimposed scatter plots of **b** HN dp6 and HP dp6, **d** HN dp10 and HP dp10, and **f** HN dp20 and HP dp20. Scatter plot of the mixture of **c** HN dp6 and HP dp6, **e** HN dp10 and HP dp10, and **g** HN dp20 and HP dp20. Discrimination between HP and HN oligosaccharides in each size class is observed. All data were recorded in 4 M LiCl, 25 mM HEPES buffer and 1.0 mM EDTA at pH 7.5, 20.0 °C, and at the bias voltage of +60 mV. The concentrations in the mixture for HN and HP dp6 were 50 μM and 10 μM, respectively; HN and HP dp10 were 50 μM and 25 μM, respectively; and. HN and HP dp20 were 10.5 μM and 3.5 μM, respectively. In order to avoid pore closure, lower concentrations were used for the dp20 chains. Source data for this figure are provided as a Source Data file.

In addition to size heterogeneity, the diversity of sulfation patterns is also an analytic barrier. To investigate the effect of sulfation on the translocation behavior of the GAG oligosaccharides, heparin oligosaccharides were first compared with their unsulfated counterparts obtained from heparosan (HN). HN consists of [−4-*N*-acetylglucosamine- α1,4- glucuronic acid- β1-]_n_ disaccharide units, which can thus be considered as a fully desulfated *N*-acetylated HP with identical glycosidic linkages to HP (the uronic unit being GlcA in HN and GlcA/IdoA in HP) (Figs. 1 and 4a).

A striking difference was observed between HP and HN oligosaccharides on the scatter plots, which show two quite distinct event populations for dp6 (Fig. 4b), dp10 (Fig. 4d) and dp20 (Fig. 4f). This discrimination is still observed for HP and HN oligosaccharides in mixture for each size class (Fig. 4c, e, g). Some scattering of translocation times of HP compared to HN can be noted, which can be explained by some deviation of the main structure of the HP trisulfated disaccharide unit (resulting from some variation in the degree and pattern of sulfation). HN is a regular unsulfated GAG and thus does not have this drawback. The resolution of the HP and HN oligosaccharides into two distinct populations results from different blocking currents: HP oligosaccharides led to greater signal extinction, suggesting the importance of sulfate groups in the total volume occupied by the molecule in the pore lumen. It should be noted that other structural parameters could also be involved (such as the hydration state of the pore and/or the analyte in the pore, hydrophobic interactions, hydrogen bonding, ion binding to the analyte, etc.), in particular due here to the sulfate groups that can generate ionic interactions with the pore. However, if the latter also made a significant contribution to translocation, we would also see its effect on the translocation time. But the observation of almost comparable T_t_ values for HP and HN oligosaccharides shows that this interaction effect plays little, and that the dominant effect is that of the occupied volume.

Translocation times of HP and HN oligosaccharides shown in Fig. 4 are as follows: HP dp6: 23 ± 20 µs, HN dp6: 50 ± 15 µs, HP dp10: 86 ± 10 µs, HN dp10: 71 ± 10 µs, HP dp20: 391 ± 17 µs and HN dp20: 297 ± 20 µs. The higher anionic charge of the HP oligosaccharides due to the sulfate groups can promote their faster translocation as observed for the small oligosaccharide dp6. However, the larger monomer size in HP oligosaccharides should enhance the steric and electrostatic interactions within the pore. The impact of these interactions on transit time could be greater than that of the anionic charge as observed for dp20 oligosaccharides. Indeed HP dp20 have a longer translocation time than HN dp20. The two effects of anionic charge and steric hindrance are almost counterbalanced in the case of HP dp10 and HN dp10 leading to almost similar translocation times but remain discernible on the basis of their *I*_b_/*I*_0_ values.

With the mixture of HP and HN oligosaccharides (Fig. 4e), a more intense peak is observed for HP than for HN due to the higher translocation frequency of the HP oligosaccharides than that of the HN oligosaccharides (at the difference of Fig. 4d in which the analysis of HP and HN dp10 was performed individually, and then their scatter plots were determined for a similar number of recorded events and were not rescaled by event frequencies). Despite higher concentrations of HN oligosaccharides to compensate for the higher translocation frequency of HP oligosaccharides, the effect of this higher frequency could not be fully mitigated.

To probe the regioselective influence of the sulfate groups on the translocation properties, selectively desulfated heparin dp10 oligosaccharides, namely 2-*O*-desulfated HP dp10, 6-*O*-desulfated HP dp10, *N*-desulfated HP dp10 and *N*-desulfated re *N*-acetylated HP dp10 (Supplementary Fig. 5), were analyzed. All these regioselectively desulfated HP decasaccharides showed distinct translocation behavior from that of HP dp10 (Supplementary Fig. 6). They all exhibited higher normalized blockade current values (shallower events), confirming the role of the sulfate groups in confinement properties via the occupied space. In addition, the translocation times of these selectively desulfated heparins have shifted to shorter times even though the partial lack of sulfate groups resulted in a decrease of the charge state of the molecule. Interestingly, the translocation time of the *N*-desulfated re *N*-acetylated HP dp10 most closely approximates that of HP dp10, although it remains shorter. Despite their similar volumes and the higher anionic charge of HP dp10, the translocation of HP dp10 is slower. These results indicate that the translocation behavior of HP dp10 through AeL nanopore is more influenced by the space occupied by the sulfate groups rather than by the anionic charge. Note the greater impact of 6-*O*- and *N*- desulfation, consistent with their exo-cyclic position on the sugar ring compared to the 2-*O*-sulfate group.

To examine whether the ability of the nanopore detection system to differentiate different levels of sulfation can be exploited to monitor biological reactions modifying the sulfate content, the enzymatic regioselective 6-*O*-desulfation of HP dp 10 and HP dp20 catalyzed by the sulfatase HSulf-2 was investigated (Supplementary Notes 2 and 3, and Supplementary Figs. 7 and 8). The same shift of the translocation times to shorter times after enzymatic desulfation were observed in both cases (with a larger shift for HP dp20 compared to HP dp10), in accordance with the above observations using commercially purchased regioselectively desulfated heparins.

### Osidic bond

**Fig. 5:**
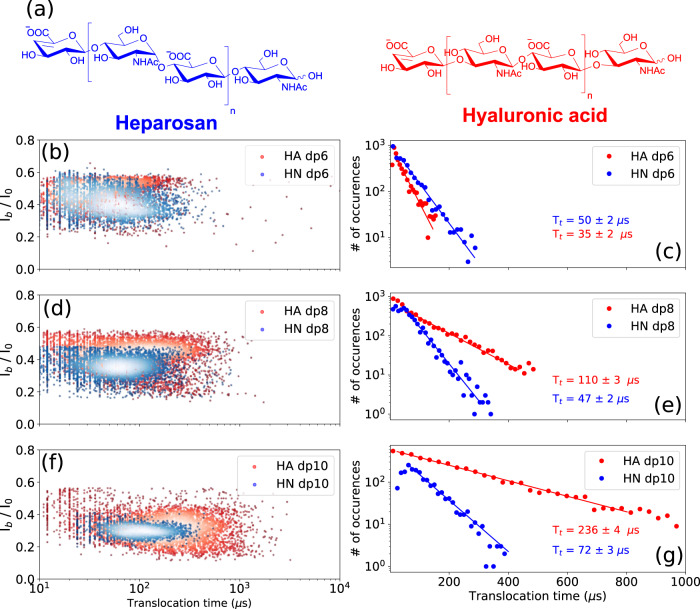
Effect of the osidic bond on the translocation behavior of the GAGs. **a** Structures of the heparosan (HN) and hyaluronic acid (HA). Superimposed scatter plots of the **b** HA dp6 and HN dp6, **d** HA dp8 and HN dp8, and **f** HA dp10 and HN dp10. Superimposed *T*_t_ histograms of the **c** HA dp6 and HN dp6, **e** HA dp8 and HN dp8, and **g** HA dp10 and HN dp10. A noticeable difference in translocation time is observed between HA and HN oligosaccharides. HA oligosaccharides show a much steeper ramp in increase of the *T*_t_ as a function of size than HN oligosaccharides. The time distributions were fitted to a mono-exponential function leading to the timescales given on the corresponding graphs. All data were recorded in 4 M LiCl, 25 mM HEPES buffer and 1.0 mM EDTA at pH 7.5, 20.0 °C, and at the bias voltage of +60 mV. Source data for this figure are provided as a Source Data file.

To assess the effect of the osidic bond on the translocation behavior of GAGs, GAG oligosaccharides composed of similar building blocks but linked together by dissimilar osidic linkages were analyzed. For this purpose, dp6, dp8 and dp10 oligosaccharides were prepared from heparosan (HN) and hyaluronic acid (HA) composed of [−4-*N*-acetylglucosamine- α1,4- glucuronic acid- β1-]_n_ and [−3-*N*-acetylglucosamine- β1,4- glucuronic acid- β1-]_n_ disaccharide units, respectively (Fig. 5a).

A drastic difference in translocation time was observed between HA and HN oligosaccharides, with HA oligosaccharides showing longer dwell times (Fig. 5c, e, g). Calculated translocation times are for HA oligosaccharides: HA dp6: 35 ± 15 µs, HA dp8: 110 ± 15 µs and HA dp10: 237 ± 15 µs, and for HN oligosaccharides: HN dp6: 50 ± 15 µs, HN dp8: 46 ± 10 µs and HN dp10: 71 ± 10 µs. The differences in *T*_t_ between HA and HN in each size class increase with oligosaccharide size, and HA oligosaccharides show a much steeper rate of increase in *T*_t_ than HN oligosaccharides. The large difference observed between the dp10 oligosaccharides suggests that HA adopts conformations inducing more interactions within the AeL nanopore. In addition, much more dispersed event clouds are observed for HA oligosaccharides compared to HN oligosaccharides (Fig. 5b, d, f). This observation can be related to data from previous studies reporting the coexistence of various HA conformations depending on its environment, as well as interconversion between different HA conformations^35–38^. In order to ascertain this hypothesis, and to make sure that the wider signal distribution observed for HA oligosaccharides is not a simple artefact of an impure sample, HA and HN dp6, dp8, and dp10 oligosaccharides were analyzed and compared with size exclusion chromatography system using two columns in series (Supplementary Fig. 9). Almost similar chromatographic separation is observed between the different sizes of oligosaccharides in each class, whereas narrow event distributions for HN oligosaccharides and broad distributions for HA oligosaccharides were observed in the nanopore data. This shows that the broad distribution of translocation events for HA oligosaccharides does not originate from impurities, and is, in fact, due to the coexistence of various conformations. Moreover, in each size class, no distinction between HN and HA oligosaccharides can be seen by chromatography. However, using the nanopore technique, we see a huge distinction in terms of translocation times and the width of the event distributions, demonstrating the strength of this technique. A high concentration of LiCl was used in our study, and further investigation is needed to determine the influence of the concentration and nature of salt on HA translocation pattern. Under our conditions, discrimination of HA and HN oligosaccharides of two different sizes in a mixture is feasible as observed for the mixture of HA dp6 and HN dp10 (Supplementary Fig. 10). However, due to the dispersion of the event population for HA oligosaccharides, it is difficult at this stage to distinguish them from HN oligosaccharides of various lengths in a mixture. Overall, these results show that differences in α/β anomery and 1,4/1,3 osidic bonds can be thus detected by nanopore sensing.

### Epimerization

**Fig. 6:**
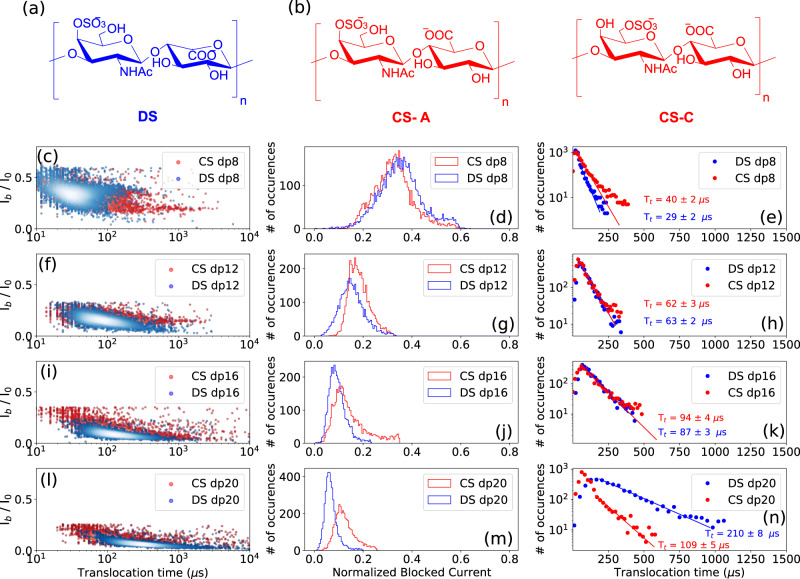
Probing the effect of GlcA / IdoA epimerization on the translocation behavior of the GAGs. Structure of **a** dermatan sulfate (DS) and **b** chondroitin sulfate (CS) A and C. Superimposed scatter plots of the **c** DS dp8 and CS dp8 **f** DS dp12 and CS dp12, **i** DS dp16 and CS dp16, and **l** DS dp20 and CS dp20. Superimposed *I*_b_ histograms of the **d** DS dp8 and CS dp8 **g** DS dp12 and CS dp12, **j** DS dp16 and CS dp16, and **m** DS dp20 and CS dp20. Superimposed *T*_t_ histograms of the **e** DS dp8 and CS dp8, **h** DS dp12 and CS dp12, **k** DS dp16 and CS dp16, and **n** DS dp20 and CS dp20. Slightly deeper current blockades are observed for DS oligosaccharides suggesting a larger spatial volume of DS compared to CS oligosaccharides. While DS dp8 presents a slightly shorter *T*_t_ compared to CS dp8, DS and CS dp12 and dp16 oligosaccharides show comparable dwell times, and a longer *T*_t_ is observed for the DS dp20 compared to CS dp20. The time distributions were fitted to a monoexponential function leading to the timescales given on the corresponding graphs. All data were recorded in 4 M LiCl, 25 mM HEPES buffer and 1.0 mM EDTA at pH 7.5, 20.0 °C, and at the bias voltage of +60 mV. Source data for this figure are provided as a Source Data file.

Determining the epimerization of uronic acid residues in GAG chains is one of the most challenging tasks in GAGs analysis, with the knowledge that this subtle difference in the hexuronic acid stereochemistry results in distinct biological properties. The ability of the AeL nanopore to distinguish between glucuronic acid (GlcA)/iduronic acid (IdoA) epimers was investigated here by analyzing the translocation of sulfated oligosaccharides from dermatan sulfate (DS) containing IdoA and chondroitin sulfate (CS) containing GlcA. DS consists of [−3-*N*-acetylgalactosamine 4S- β1,4- iduronic acid- β1-]_n_ disaccharide units (Fig. 6a), and CS consist of [−3-*N*-acetylgalactosamine 6 S or 4S- β1,4- glucuronic acid- β1-]_n_ disaccharide units (Fig. 6b). The *N*-acetylgalactosamine residues are 4-*O* (CS-A) or 6-*O* (CS-C) sulfated in CS oligosaccharides, and 4-*O*-sulfated in DS oligosaccharides (Fig. 6a, b).

Slightly deeper current blockades, getting more significant with increasing the oligosaccharide size, were observed for DS oligosaccharides suggesting a larger spatial volume of DS compared to CS (Fig. 6d, g, j, m). Regarding translocation times, while DS dp8 presents a slightly shorter *T*_t_ than CS dp8 (Fig. 6e), comparable dwell times are measured for the DS and CS dp12 and dp16 oligosaccharides shown in (Fig. 6h, k), and a longer *T*_t_ is observed for the DS dp20 than for CS dp20 (Fig. 6n), suggesting a configuration of the DS dp20 molecule that induces more interactions within the pore. IdoA residues can adopt three different conformations, namely ^1^C_4_ (chair), ^2^S_0_ (skew boat) and ^4^C_1_ (chair) with the possibility of inter-conversion, which allows a great flexibility of the DS chains, while CS chains are less flexible since GlcA exists only in the ^4^C_1_ (chair) conformation. We can assume that the longer DS oligosaccharide feeds a greater diversity of configurations, which leads to a higher probability of interaction within the pore resulting in longer translocation times. Translocation events appear more dispersed for CS oligosaccharides than for DS oligosaccharides, which may be due to the fact that the chondroitin sulfate oligosaccharides used in our experiments are mixtures of CS-A and CS-C. From these observations, it appears that the nanopore detection technique is able to distinguish two classes of GAGs differentiated by a structural feature as fine as epimerization.

### Building block

Hyaluronic acid and chondroitin sulfate are two main families of GAGs with the same alternating glycosidic bonds β1-4/β1-3 between the units, but different types of building blocks. While HA has *N*-acetylglucosamine unit in its building block, CS has *N*-acetylgalactosamine 6 S or 4 S (Supplementary Fig. 11). From the translocation event plots and *I*_b_ histograms of dp6, dp8, and dp10 oligosaccharides from HA and CS **(**Supplementary Fig. 11), we observe that CS oligosaccharides exhibit deeper translocation events than those from HA, most likely due to the larger spatial volume occupied by sulfated building blocks in CS. CS and HA oligosaccharides are also differentiated by their translocation time, with HA oligosaccharides moving much more slowly due to their lower anionic charge. As the size of the oligosaccharides increases, the difference between the translocation times of HA and CS becomes greater. Therefore, although the HA and CS oligosaccharides share the same osidic linkage pattern, they exhibit distinct nanopore signatures, which may be explained by the sensing capabilities of the AeL nanopore with respect to the isomerization and functional groups of the acetylhexosamine unit.

## Discussion

**Fig. 7:**
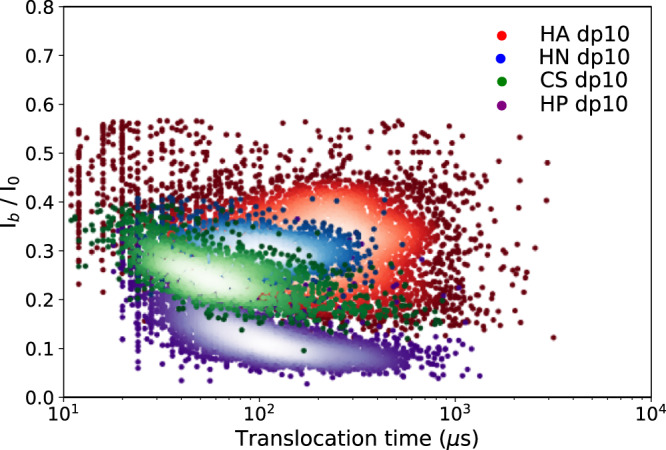
Superimposed scatter plot of events observed with dp10 oligosaccharides of hyaluronic acid (HA), heparosan (HN), chondroitin sulfate (CS), heparin (HP). It shows the differences between the various families of oligosaccharides observed in this study. They vary by their osidic bond (HA *vs* HN), building block (HA *vs* CS) or degree of sulfation (HP *vs* HN). The timescales of translocation and the blocked current are different for each species and the event cluster are distinguishable, establishing a fingerprint of these oligosaccharides. All the experiments were performed using similar experimental conditions: 4 M LiCl, 25 mM HEPES buffer and 1.0 mM EDTA at pH 7.5, 20.0 °C, and at the bias voltage of +60 mV. Concentrations of HN and HA dp10 were 100 μM, and HP and CS dp10 were 43 μM. Source data for this figure are provided as a Source Data file.

To our knowledge, this is the first study to explore the ability of a biological nanopore to detect different sequences of GAGs oligo- and polysaccharides. While the nanopore detection method is already widely applied for nucleic acid sequencing and more recently for peptide discrimination, it had not yet been explored for the very high structural complexity of GAGs sequences. Compared to nucleic acids and proteins, GAGs show an unparalleled diversity of sequences with different combinations of sulfation, epimers of the uronic acid unit and isomers of the *N*-acetyl hexosamine unit, and osidic linkages. Our study shows that the AeL nanopore, thanks to its confinement properties, is able to detect different GAGs sequences on the basis of each of these structural determinants. In particular, characteristic fingerprints could be obtained for various GAGs based on the sulfation and epimerization of the osidic units (Fig. 7). These structural elements distributed along the sequence of GAGs represent an informational content guiding the functional properties of GAGs, and the results obtained here open the way to their deciphering at the single molecule level. Unlike solid nanopores, biological nanopores have a diameter of the same scale as many single molecules and molecular building blocks, so that sequential reading of the monomeric composition and detection of fine modifications of the linear oligosaccharide molecule can be considered. This desired molecular resolution is not yet achieved in this study since the disaccharide units are not detectable due to their too fast translocation speed. However, this goal should be achievable through the ability of biological nanopores to be tailored with angstrom precision using protein engineering techniques, as previously reported for the successful detection and discrimination of single amino acids^22^. In addition, a recent work reported the ability of the AeL nanopore to directly detect a single amino acid molecule without any modification or labeling^39^. Thus, it can be anticipated that monosaccharide detection should be achievable by regulating the characteristic interaction between the monosaccharide unit and the designed AeL nanopore.

In addition to determining the different structural elements of oligosaccharides, nanopore analysis also highlights the impact of these elements on structural organization. Thus, a difference as subtle as that of the osidic bond between the constituent units, as with HA and HN, leads to distinct signatures, indicating a very different occupation of the space in the nanopore, and therefore reflecting very distinct conformational organizations of the confined molecules. Finally, the detection method proves to be useful to detect fine structural modifications generated by enzymatic action, as illustrated here by the regioselective 6-*O*- desulfation catalyzed by the sulfatase HSulf. This opens the way to fine analysis at the single molecule level of the regioselectivity of enzymes acting on GAGs. The single molecule level of detection, which requires only a minute amount of analyte, and label-free detection are very valuable features for GAGs monitoring in the biological and medical diagnosis context.

In this study, GAGs differing in one main structural attribute (e.g. sulfation) were compared. However, GAGs often show a spread of more than one structural attribute throughout their chain. In addition, sulfation and epimerisation of GAGs generally vary along individual GAG chains. Therefore, this study, which is an essential step on the way to the identification of GAGs, also highlights the need for further studies and progress to achieve the comprehensive identification of individual GAG molecules with their heterogeneities. We are considering several avenues to accomplish this goal of sequencing individual molecules. These could involve, among others, better control of the translocation rate using molecular motors as already exists for DNA sequencing, the use of GAG-specific enzymes combined with nanopore analysis, and improved data analysis capabilities through comprehensive signal processing of the recorded electrical traces and in-deep data mining. It should be noted that the same scientific barriers also had to be overcome for DNA sequencing and, more recently, for protein sequencing, bearing in mind that nucleic acids can be amplified to purity, whereas the pool of GAG molecules obtained from natural sources is never uniform. This difference poses an important additional challenge in GAG analysis. Advances in recent years in the nanopore analysis of these two classes of biological macromolecules are based on translocation control allowing the sequential reading of the molecules during its translocation and on fingerprinting obtained by enzymatic digestion, followed by the identification the fragments. For example, depolymerization of heparan sulfate with the sulfated sequence-specific heparinase I releases the unsulfated domains, and treatment with unsulfated sequence-specific heparinase III releases the sulfated domains. Nanopore fingerprinting of these two types of enzyme products will allow the determination of the organization of the HS domains as a function of tissue or pathophysiological status. The use of regioselective sulfatases, such as the 6-*O*-sulfatase tested in this study will be useful in deciphering the sulfation patterns. Whether one or the other method is used, it requires an improvement of the signal resolution. We are already working on other event parameters that could be added to the event duration and normalized blocked current to go beyond the level of statistical resolution achieved in this study and reach single-molecule scale identification. The use of machine learning on a database of nanopore signatures of known molecules will help in the structural identification within this complex parameter space.

## Methods

### Materials

Heparin, regioselectively modified heparin, chondroitin sulfate, dermatan sulfate and hyaluronic acid oligosaccharides as well as heparin 11 kDa were purchased from Iduron (Manchester, UK). These commercial oligosaccharides were prepared by the provider following well-established depolymerization methods using the specific enzymes heparinases, chondroitinases, and hyaluronidase, and purified by high-resolution gel filtration. Each size class of oligosaccharide is substantially homogeneous in molecular size as demonstrated by size exclusion chromatography analysis; however, as indicated by the provider, it may contain structures that can vary a little bit in content and pattern of sulfation, that is consistent between various dpn. Preparation methods of the different oligosaccharides studied, and their main structural features are summarized in Supplementary Table 1. Mass spectra of the regioselectively modified heparin oligosaccharides are presented in Supplementary Fig. 12 and Supplementary Data file 1. Heparin 5 kDa was obtained from Neoparin Inc., and heparin 16 kDa was from Celsus Laboratories Inc. (Cincinnati, OH, USA). The low molecular weight heparin enoxaparin Lovenox® was from Sanofi-Aventis (Bridgewater, NJ). Heparosan and derived oligosaccharides were produced by the multi-enzymatic complex synthase of heparosan and endo-lyase expression in metabolically engineered *E. coli* K5^40^. Briefly, recombinant *E coli* DH1 expressing *kfiABCD* and *elma* genes encoding for the multi-enzymatic complex synthase of heparosan and endo-lyase of *E coli* K5 was grown in 2 L bioreactor. The medium was composed of glycerol (17.5 g L^−1^), NH_4_H_2_PO_4_ (7 g L^−1^), KH_2_PO_4_ (7 g L^−1^), MgSO_4_·7H_2_O (1 g), thiamine·HCl (4.5 mg L^−1^), trace mineral solution (7.5 mL L^−1^), citric acid (0.5 g L^−1^), KOH (2 g L^−1^). The trace mineral stock solution contained: nitrilotriacetate (70 mM), ferric citrate (7.5 g.L^−1^), MnCl_2_·4H_2_O (1.3 g L^−1^), CoCl_2_·6H_2_O (0.21 g L^−1^), CuCl_2_·2H_2_O (0.13 g L^−1^), H_3_BO_3_ (0.25 g L^−1^), ZnSO_4_·7H_2_O (1.2 g L^−1^), Na_2_MoO_4_·2H_2_O (0.15 g L^−1^). The pH was kept at 6.8 by ammoniac addition and temperature was kept at 33 °C until total consumption of glycerol. Then, temperature was dropped at 28 °C and a glycerol feeding at 5 mL h^−1^ was started and 0.2 mM IPTG was added allowing the recombinant gene expression. After 48 h, the culture was stopped and HN oligosaccharides were recovered from the cell pellet after centrifugation and heating at 100 °C for 40 min. They were purified by anion-exchange chromatography on Dowex 1 × 4–400 on formiate form upon gradient elution with 0–1 M ammonium formiate. A second step of purification by size exclusion chromatography on Superdex was performed if required. 6-*O*-desulfated HP dp10 and dp20 was formed by enzymatic desulfation catalyzed by the sulfatase HSulf-2 at 37 °C in 50 mM Tris buffer, 10 mM MgCl_2_, pH 7.5, containing HP oligosaccharides in a 100:1 (v/v) oligosaccharides/sulfatase ratio^41^. TPCK-Trypsin (from bovine pancreas) was obtained from Thermo Fisher Scientific (Rockford, IL, USA), and lithium chloride, EDTA (ethylenediaminetetraacetic acid) and HEPES (4-(2-hydroxyethyl)−1-piperazineethanesulfonic acid), hexane and hexadecane from Sigma-Aldrich (Saint-Quentin Fallavier, France). Ultra-pure water (18.2 MΩ) was obtained from a Milli-Q purification system (Millipore).

### Single-channel recording experiments

Nanopore experiments were performed using a horizontal lipid bilayer Teflon device^42^. Briefly, the setup is made of two chambers (i.e., the *cis* and *trans* chambers) connected to each other by a sub-mm inner diameter capillary. The lipid bilayer (with a typical capacitance of 10 ± 1 pF) was formed by painting a film of 1,2-diphytanoyl-sn-glycero-3-phosphocholine (Avanti Polar Lipids, Alabaster, AL, USA) over a conical aperture of 20–30 μm in diameter separating the cis and trans chambers. To apply a fixed voltage and measure the ionic current, two Ag-AgCl electrodes (1.0 mm diameter, Aldrich, Milwaukee, WI, USA) were installed in the *cis* and *trans* chambers filled with 100 μL of buffer containing 4 M LiCl, 25 mM HEPES and 1.0 mM EDTA at pH 7.5. The whole Teflon cell was fixed in a temperature-controlled copper enclosure in order to control the buffer temperature. A Peltier device controlled by a temperature controller (Newport 3040, Irvine, CA, USA) in conjunction with a heating circulator (CORIO CD-BT5, Julabo USA Inc., Allentown, PA, USA) around the copper enclosure base was used to set the temperature. The entire setup was placed within a grounded faraday cage to electrically shield it from electromagnetic interference.

Recombinant wild-type pro-aerolysin was synthetized using the following procedure as previously reported^19^. We transformed *Escherichia coli* BL21 strain with pET22b-proAL plasmid containing pro-aerolysin sequence. This plasmid allowed the induction of pro-aerolysin production with IPTG (1 mM final concentration) and the periplasmic localization of the recombinant protein. The periplasm was extracted with an osmotic shock and pro-aerolysin was furthermore purified by affinity chromatography using the C-terminal his-tag of the recombinant protein (His SpinTrap minicolumns, GE Healthcare Life Science). Pro-aerolysin binding to the Ni-Sepharose was made with 100 mM Tris-HCl pH 7.4 and 50 mM imidazole, and after three washing steps, elution was made with 100 mM Tris-HCl pH 7.4 and 500 mM imidazole. The recombinant pro-aerolysin purity was determined by SDS-polyacrylamide gel electrophoresis and Coomassie blue staining, to 99 ± 1% (w/w). Concentration was calculated by absorbance at 280 nm. The pro-aerolysin monomers were stored at 4 °C at a final concentration of 0.1 g/L.

Recombinant wild-type pro-aerolysin was activated by trypsin digestion (0.6 μM trypsin final concentration) during 15 min at room temperature as previously reported^19^ to eliminate the pro-peptide sequence. After the formation of the lipid bilayer, activated aerolysin was added to the *cis*-chamber at 0.25 μg mL^−1^ final concentration. The insertion of a single nanopore in the lipid bilayer was identified as a step-jump of the transmembrane ionic current. Single AeL nanopore generated an ionic current of 58 ± 3 pA under an applied voltage of +60 mV (electrode in the *cis* chamber was grounded) at a temperature of 20 ± 1 °C.

The current traces were amplified and measured using a patch-clamp amplifier (MultiClamp 700B, Molecular Devices, San Jose, CA, USA) with a CV-7B headstage and the signals were low-pass filtered at 50 kHz using a 4-pole Low-Pass Butterworth filter (Krohn-Hite 3361, Brockton, MA, USA). The signal was digitized at 1 MHz/12 bits using a DAQ card (NI DAQ PCI 6259 M serie, National Instruments, Austin, TX, USA). All data acquisition and measurement automation were performed using an in-house developed National Instruments LabView. Statistical analysis was carried out using Igor Pro 8 software (WaveMetrics, Lake Oswego, OR, USA). The graphs were made using Python 3 with the Matplolib and SciPy libraries. Detailed description of the data analysis is presented in the supporting information (Supplementary Note 4).

### Statistics & reproducibility

The experiments were not randomized. No statistical method was used to predetermine sample size.

### Reporting summary

Further information on research design is available in the Nature Research Reporting Summary linked to this article.

## Supplementary information

Supplementary Information

Descriptions for additional Supplementary File

Supplementary Data 1

Reporting Summary

## Data Availability

Data files supporting this study have been deposited in the Zenodo database under 10.5281/zenodo.6861919. Source data for Figs. 2 to 7 and all Supplementary Figures are provided with this manuscript. Source data are provided with this paper.

## Source data

Source Data

## Code availability

All analysis and graphs plotting custom code and algorithms used in this study are available at 10.5281/zenodo.6861919.
