# Supplementary material for: Comprehensive structural assignment of glycosaminoglycan oligo- and polysaccharides by protein nanopore
Source: Nat Commun. 2022 Aug 30;13:5113. doi: 10.1038/s41467-022-32800-4 (PMC9427770; doi:10.1038/s41467-022-32800-4)
Supplement: Supplementary file 1 — Supplementary Information [file 41467_2022_32800_MOESM1_ESM.pdf]

Supplementary Information for:

Comprehensive structural assignment of glycosaminoglycan oligo- and polysaccharides by protein nanopore

Parisa Bayat[1], Charlotte Rambaud[1], Bernard Priem[2], Matthieu Bourderioux[1], Mélanie Bilong[1], Salomé Poyer[1], Manuela Pastoriza-Gallego[3], Abdelghani Oukhaled[3], Jérôme Mathé[1*], Régis Daniel[1*]

[1]Université Paris-Saclay, Univ Evry, CNRS, LAMBE, 91025, Evry-Courcouronnes, France
[2]CNRS, CERMAV, University Grenoble Alpes, 38000 Grenoble, France
[3]CY Cergy Paris Université, CNRS, LAMBE, 95000 Cergy-Pontoise, France

[*]Correspondence to:
Régis Daniel, regis.daniel@univ-evry.fr
Jérôme Mathé, jerome.mathe@univ-evry.fr

Supplementary Note 1

Optimization of the voltage, and type and concentration of the electrolyte: experimental parameters such as the applied voltage and type and concentration of the electrolyte were evaluated before recording the data. At voltages below +60 mV, the signal-to-noise ratio was not high enough to unambiguously distinguish true translocation events from noise, and at voltages above +60 mV, not only was the resolving power lower, but the event populations were distorted at the shorter translocation time portion of the distribution as shown in Supplementary Fig. 1. So, all the experiments were carried out at an applied voltage of +60 mV.

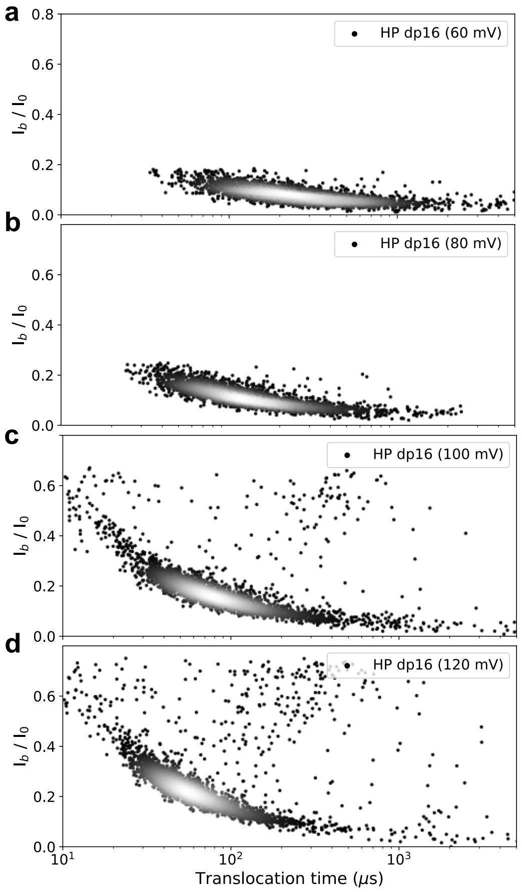

Supplementary Fig. 1 Distortion of the event population of the HP dp16 at the shorter translocation time portion of the distribution with the increase of the applied voltage, displayed when a voltage of a) 60 mv, b) 80 mV, c) 100 mV, and d) 120 mV is applied.

The LiCl salt is commonly used in nanopore analyses of biopolymers. GAGs are highly negatively charged molecules with very fast translocations through the pore. Based on the fact

that longer and deeper current blockades lead to better molecular discriminations, we searched for experimental conditions that slow down the translocation of GAGs. Previously, it was shown that the translocation time of a DNA molecule (which is also negatively charged) strongly increases when the size of the counter ion decreases from K^+ to Na^+ and Li^+ and when the concentration of the electrolyte increases.[1,2] Therefore, 3 M and 4 KCl M, as well as 4 M and 6 M LiCl were tried (**Supplementary Fig. 2**).

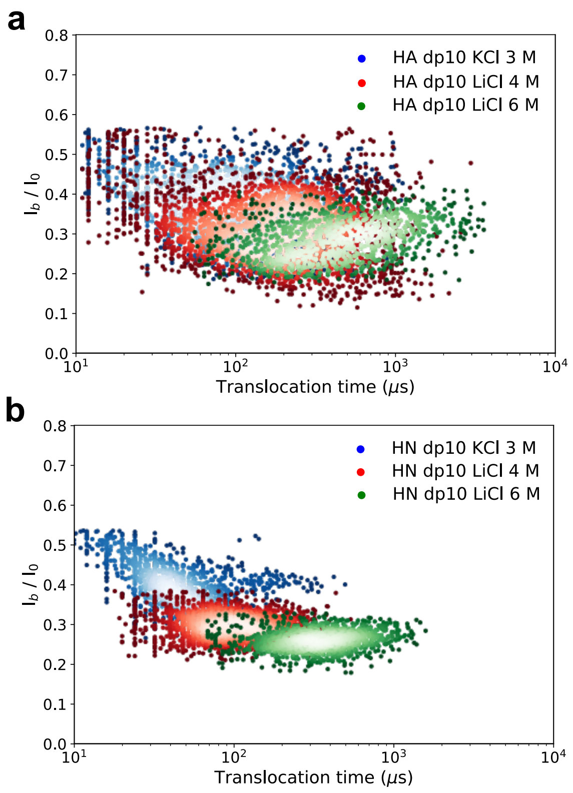

Supplementary Fig. 2 Comparing the event population using KCl 3 M (dark blue), LiCl 4 M (red) and LiCl 6 M (green) for a) HA dp10 and b) HN dp10.

From a practical point of view, it was difficult to work at KCl 4 M with our homemade horizontal Teflon lipid bilayer device, whose *cis* and *trans* compartments have a small volume (100 µL). Indeed, during the recording time, KCl precipitated, and, as a result, the salt concentration of the buffer changed. Therefore, use of LiCl with an extremely high solubility (83.2 g/100 mL (19.6 M) at 20 °C)[3] was preferred to KCl. Using 6 M LiCl, the translocation time was maximal (Supplementary Fig. 2); however, pore insertion into the bilayer was difficult. Using 3 M KCl and 4 M LiCl was practically feasible; however, slightly longer and

deeper events were observed using LiCl 4 M (Supplementary Fig. 2), and therefore we chose to work under these conditions.

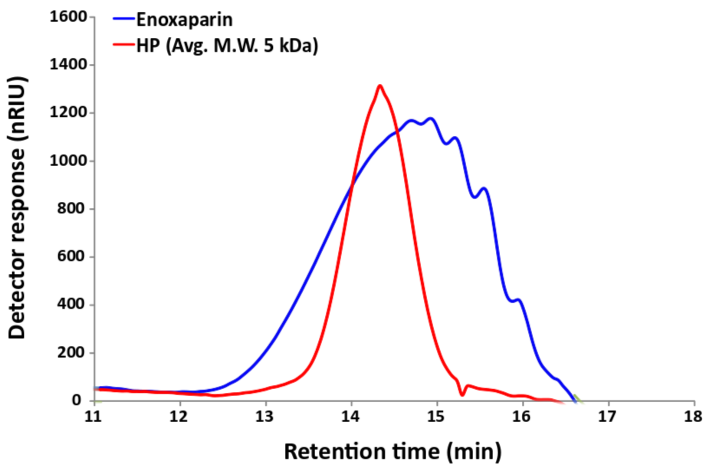

Supplementary Fig. 3 HPLC SEC analysis of Enoxaparin (blue) and HP (Avg. M.W. 5 kDa) (red). Analyses were performed using two coupled SEC columns (PL aquagel-OH 20 5 µm, 7.5 x 300 mm) in series and reflectometry detection (Agilent 1260 Infinity II). 100 mM Na_2PO_4, 300 mM NaCl, pH 7.0 was used as the eluent at a flow rate of 1 mL/min.

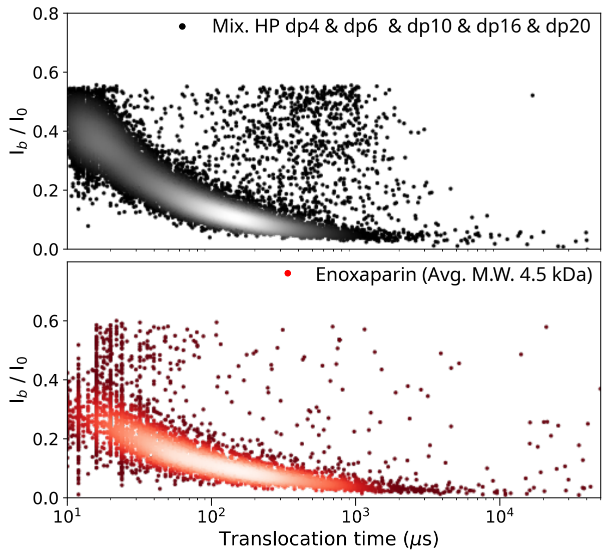

Supplementary Fig. 4 Scatter plots of (**a**) mixture of HP oligosaccharides containing dp4, dp6, dp10, dp16 and dp20 oligosaccharides, and (b) enoxaparin. The two clouds of event broaden over similar translocation timescales except at shortest times. Thus the presence of dp6-dp20 HP oligosaccharides in the enoxaparin sample can be qualitatively deduced from the plot. The data were recorded in 4 M LiCl, 25 mM HEPES buffer and 1.0 mM EDTA at pH 7.5, 20.0 °C, and at the bias voltage of +60 mV.

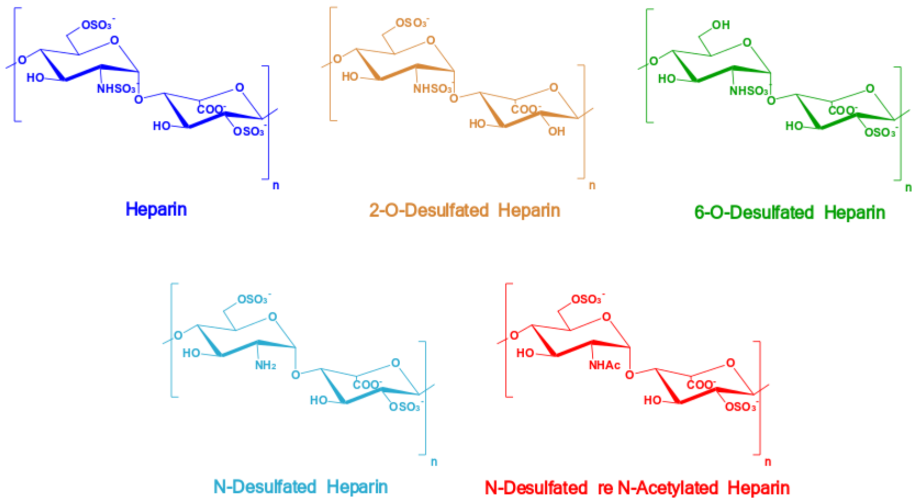

Supplementary Fig. 5 Structure of disaccharide repeating units of heparin and selectively desulfated heparins studied in this work.

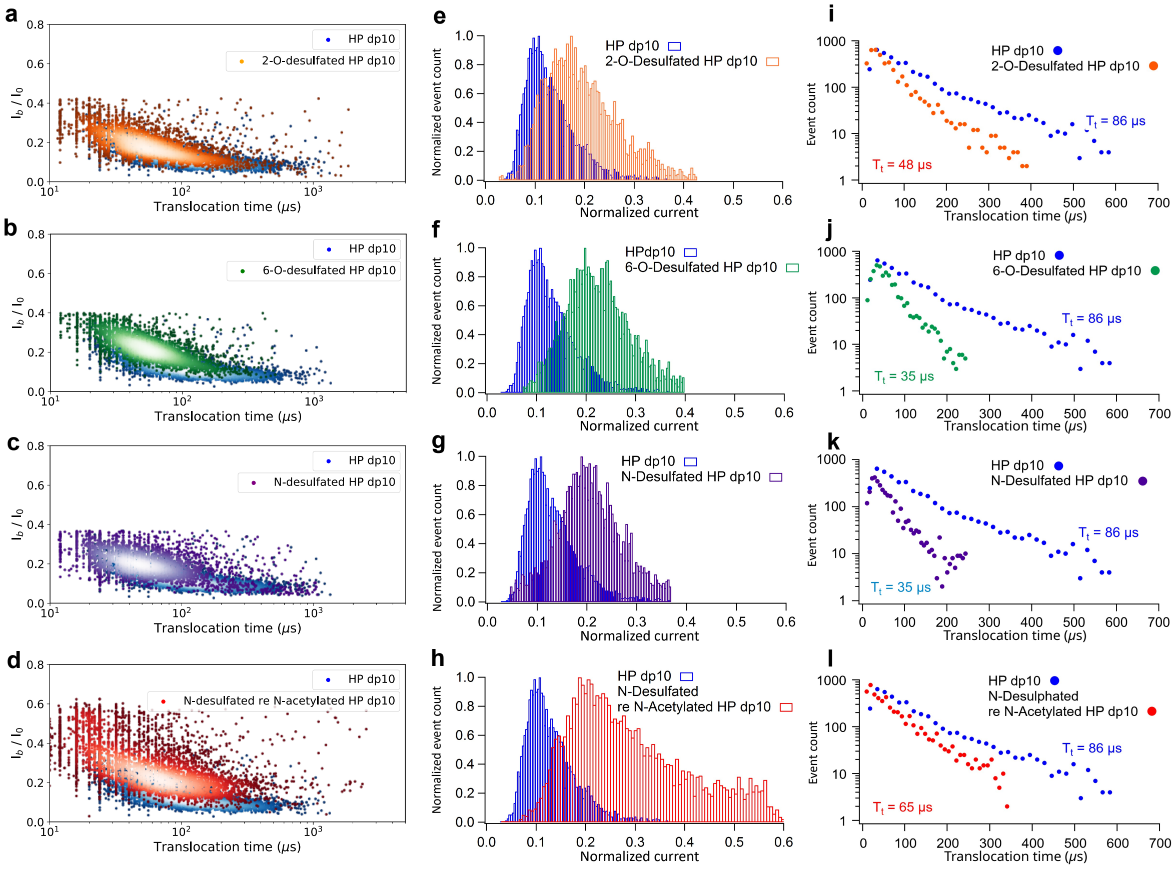

Supplementary Fig. 6 Effect of regioselective desulfation of heparin decasaccharide on its translocation behavior. Superimposed scatter plots of HP dp10 and (**a**) 2-*O*-desulfated HP dp10, (**b**) 6-*O*-desulfated

HP dp10, (**c**) *N*-desulfated HP dp10, and (**d**) *N*-desulfated re *N*-acetylated HP dp10. Superimposed I_b histograms of HP dp10 and (**e**) 2-*O*-desulfated HP dp10, (**f**) 6-*O*-desulfated HP dp10, (**g**) *N*-desulfated HP dp10, and (**h**) *N*-desulfated re *N*-acetylated HP dp10. It is noteworthy that each of the heparin decasaccharide derivatives was analyzed separately, and each time in a newly formed nanopore. All these histograms show an evident shift of the mean blocked pore current. Superimposed T_t histograms of HP dp10 and (**i**) 2-*O*-desulfated HP dp10, (**j**) 6-*O*-desulfated HP dp10, (**k**) *N*-desulfated HP dp10, and (**l**) *N*-desulfated re *N*-acetylated HP dp10. All histograms display a modification of the translocation times upon desulfation of the HP disaccharides. These histograms are fitted to exponential function (not shown for clarity). The decay of the exponential is indicated on the graph. All the regioselectively desulfated HP decasaccharides result in shallower and faster translocation events compared to HP dp10, confirming the role of sulfate group in confinement properties *via* the occupied space. All data were recorded in 4 M LiCl, 25 mM HEPES buffer and 1.0 mM EDTA at pH 7.5, 20.0 °C, and at the bias voltage of +60 mV.

Supplementary Note 2

The ability of the nanopore detection system to differentiate different levels of heparin sulfation shown on Figure S6 was exploited to monitor the enzymatic regioselective 6-*O*-desulfation of HP dp 10 and HP dp20 catalyzed by the sulfatase HSulf-2 (**Supplementary Fig. 7**). A shift of the translocation times to shorter times after enzymatic desulfation were observed (with a larger shift for HP dp20 compared to HP dp10), in accordance with the above observations on Figure S6 with regioselectively desulfated heparins.

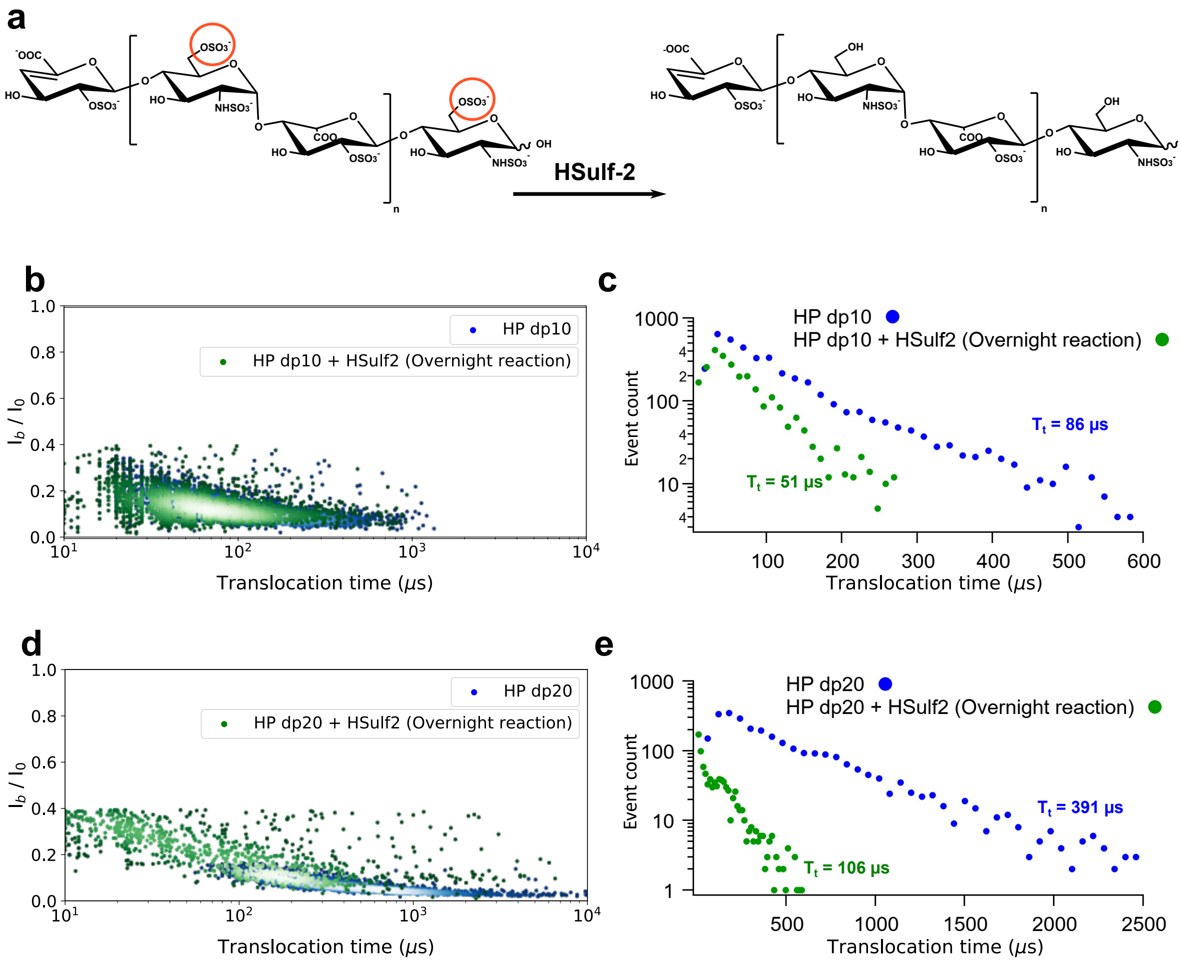

Supplementary Fig. 7 Probing the enzymatic regioselective desulfation of heparin oligosaccharides using AeL nanopore. (**a**) Regioselective 6-*O*-desulfation of HP under the action of HSulf-2 enzyme. Superimposed scatter plots (**b**) and T_t histograms (**c**) of HP dp10 and 6-*O*-desulfated HP dp10 resulted from the HSulf-2 enzymatic reaction. Superimposed scatter plots (**d**) and T_t histograms (**e**) of HP dp20 and 6-*O*-desulfated HP dp20 obtained from the HSulf-2 enzymatic reaction. For both HP dp10 and HP dp20, a shift of the translocation times towards shorter times after 6-*O*-desulfation is observed with HP dp20 undergoing a larger shift compared to HP dp10. The data were recorded in 4 M LiCl, 25 mM HEPES buffer and 1.0 mM EDTA at pH 7.5, 20.0 °C, and at the bias voltage of +60 mV.

Supplementary Note 3

It is to be noted that the action of the sulfatase enzyme on HP oligosaccharides is not complete, as shown by mass spectrometry analysis of the heparin dp10 oligosaccharide that was partially 6-*O*-desulfated upon overnight incubation with the HSulf sulfatase (**Supplementary Fig. 8)**. Indeed, HSulf sulfatase is a processive enzyme that starts its action at the non-reducing end of the oligosaccharide and then progresses towards the reducing end without, however, succeeding in hydrolyzing all the 6-*O*-sulfate groups.[35]. Therefore, compared to the oligosaccharide in **Supplementary Fig. 6b**, the oligosaccharide in **Supplementary Fig. 7b** is only partially 6-*O*-desulfated. It, therefore, occupies about the same volume in the pore lumen but carries a lower negative charge, thus not affecting the magnitude of the blockade current (for a given translocation time), in contrast to what is seen in **Supplementary Fig. 6b.**

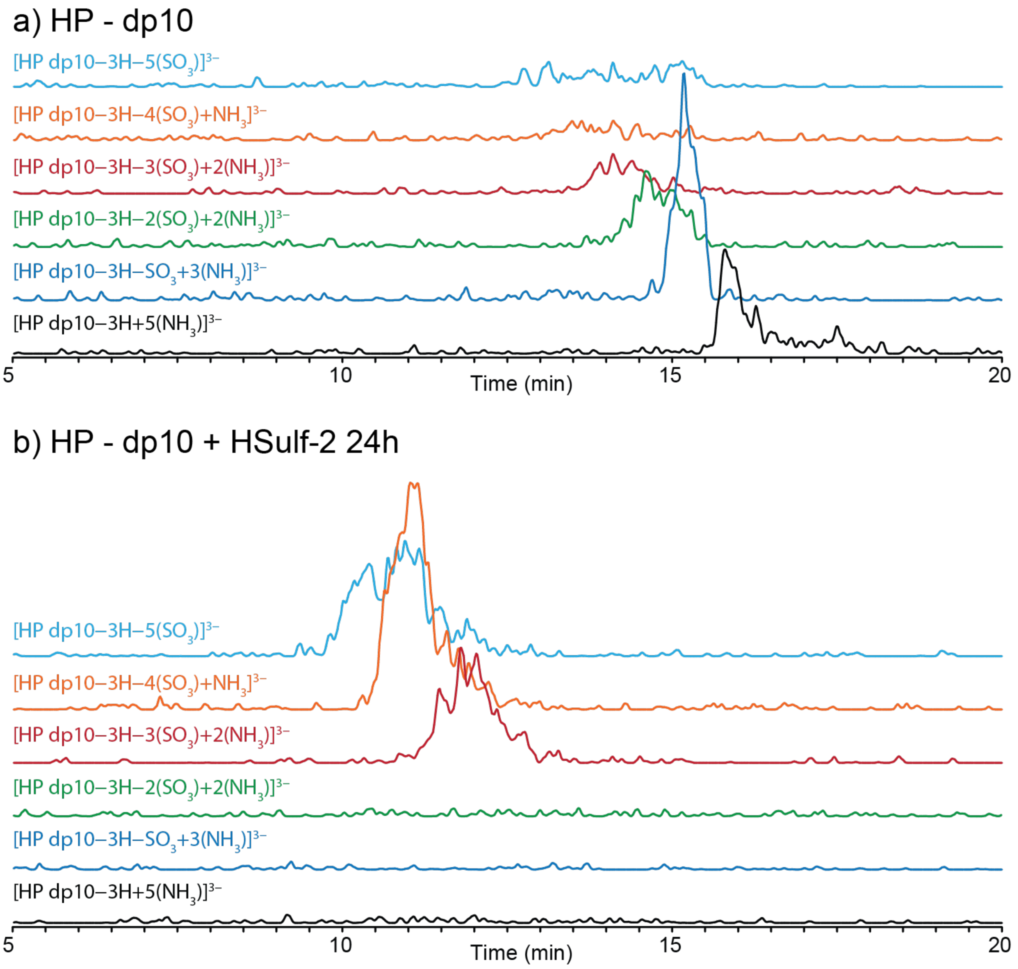

Supplementary Fig. 8 Mass spectrometry analysis of HP dp10 upon incubation with the HSulf-2 sulfatase catalyzing the regioselective 6-*O*-desulfation. MS analysis was performed by Hydrophilic liquid chromatography-MS. Extracted ion chromatograms of the triply charged (3−) ions of the HP dp10 are presented (a) before, and (b) after 24 hours of incubation with the HSulf-2 enzyme. The following sulfated species of HP dp10 were detected: the intact HP dp10 (NH_3-adduct with five 6-*O*-sulfate groups, black trace), the variably 6-*O*-desulfated HP dp10 with four (blue dark), three (green), two (red) and one (orange) remaining 6-*O*-sulfate, and the fully 6-*O*-desulfated HP dp10 (light blue). After 24 h enzymatic reaction (panel b), HP dp10 with remaining two (red) and one (orange) 6-*O*-sulfate were among the major enzyme products, highlighting the partial 6-*O*-desulfation of the starting substrate HP dp10. Minor sulfate loss occurring during electrospray ionization cannot be ruled out. HILIC-MS experiments were carried out on a Xevo-G2-S Q-TOF equipped with an electrospray source operating in negative ionization mode. The SeQuant® ZIC®-cHILIC column (3 µm, 100 Å 150 x 2,1 mm) was used with a flow rate of 300 µL/min, and oligosaccharides were eluted using a linear gradient from 40 % of A to 70 % of A in 15 minutes (solvent A: 7.5 mM ammonium formate pH 4.0, solvent B: MeCN).

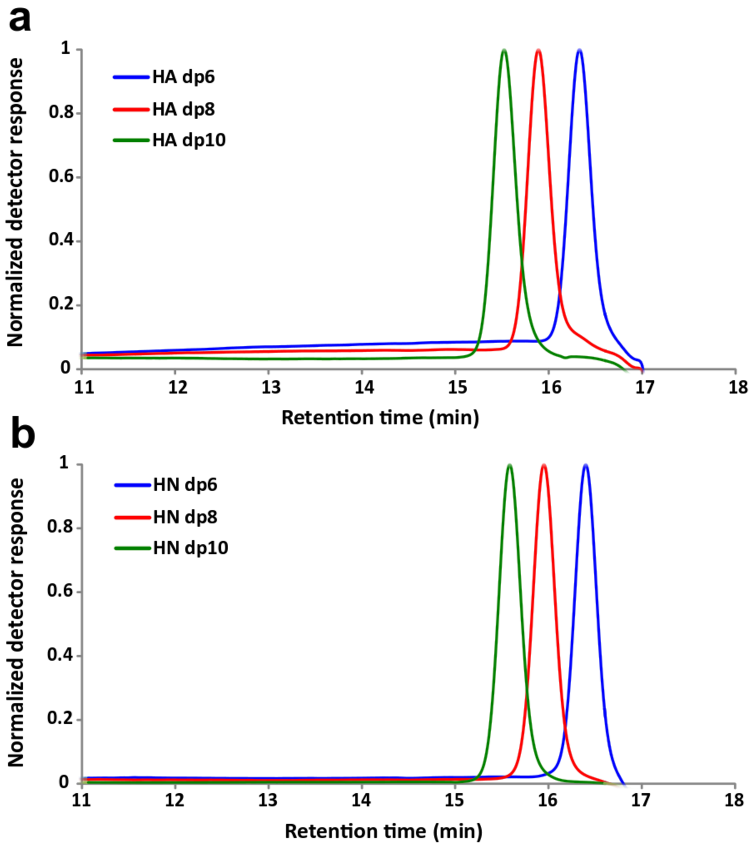

Supplementary Fig. 9 HPLC SEC analysis of (a) HA dp6, dp8 and dp10, and (b) HN dp6, d8 and dp10. Analyses were performed using two coupled SEC columns (PL aquagel-OH 20 5 µm, 7.5 x 300 mm) in series and reflectometry detection (Agilent 1260 Infinity II). 100 mM Na_2PO_4, 300 mM NaCl, pH 7.0 was used as the eluent at 1 mL/min flow rate.

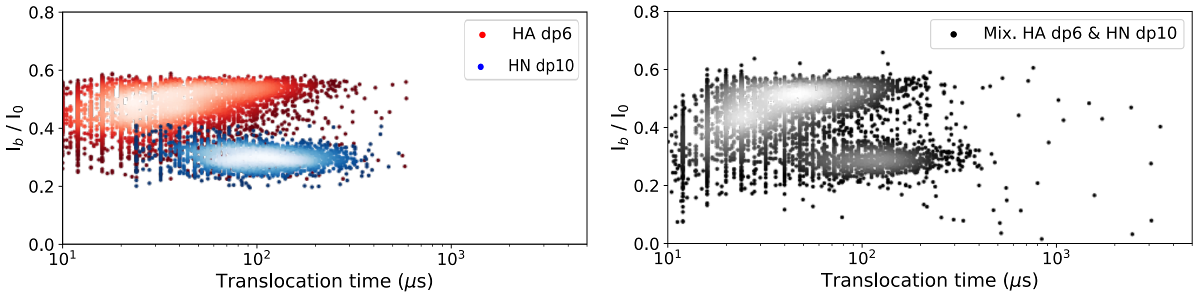

Supplementary Fig. 10 Superimposed scatter plots of individual HA dp6 and HN dp10 oligosaccharides (a) and their mixture (b). The mixture analysis displays to well define clouds of events located at the same positions than the individual HA dp6 and HN dp10. The data were recorded in 4 M LiCl, 25 mM HEPES buffer and 1.0 mM EDTA at pH 7.5, 20.0 °C, and at the bias voltage of +60 mV.

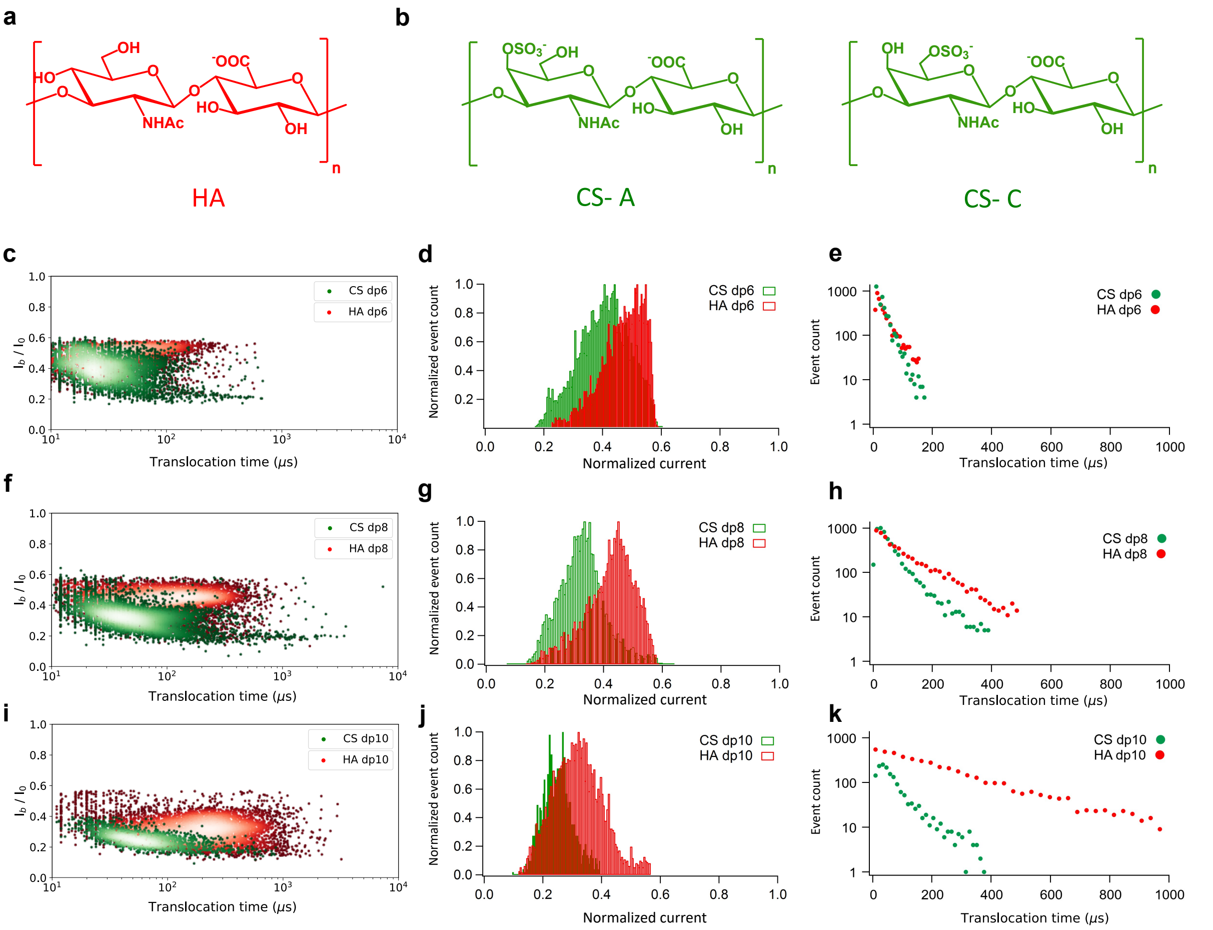

Supplementary Fig. 11 Effect of constituent building block on translocation behavior of GAGs. Structure of (**a**) hyaluronic acid and (**b**) chondroitin sulfate A and C. Superimposed scatter plots of the (**c**) CS dp6 and HA dp6, (**f**) CS dp8 and HA dp8, and (**i**) CS dp10 and HA dp10. Superimposed I_b histograms of the (**d**) CS dp6 and HA dp6, (**g**) CS dp8 and HA dp8, and (**j**) CS dp10 and HA dp10. Superimposed T_t histograms of the (**e**) CS dp6 and HA dp6, (**h**) CS dp8 and HA dp8, and (**k**) CS dp10 and HA dp10. CS oligosaccharides exhibit deeper translocation events than HA oligosaccharides due to their larger spatial volume because of the presence of the sulfate groups. In addition, higher charge states of CS oligosaccharide due to the sulfate groups leads to their faster translocation times compared to HA oligosaccharides. As the size of the oligosaccharides increases, the distinction between T_t histograms of CS and HA in each size class increases. All data were recorded in 4 M LiCl, 25 mM HEPES buffer and 1.0 mM EDTA at pH 7.5, 20.0 °C, and at the bias voltage of +60 mV.

Supplementary Table 1 Description of the commercially purchased GAGs studied in this work.

GAG family	Description
**HP oligosaccharides (Iduron)**	These oligosaccharides have been prepared by high resolution gel filtration of partial heparin lyase digestion of high quality heparin. Although the main disaccharide unit in these products is IdoUA,2S – GlcNS,6S, (approx 75%) saccharides in each size class show some variation in degree and pattern of sulfation.
**Desulphated Heparin Oligosaccharides (Iduron)**	These products have been modified by standard chemical methods to selectively remove sulfate groups from C2 of Iduronate, C6 of glucosamine or the N-sulfate of Glucosamine. The N-desulfated heparin contains the free amino group (NH^{+}_{3}); in N-desulfated re N-acetylated heparin the free amino group has been modified by acetylation.
**HA oligosaccharides (Iduron)**	These oligosaccharides have been prepared by high resolution gel filtration of partial endolyase digestion of purified Streptococcal HA.
**CS oligosaccharides (Iduron)**	These oligosaccharides have been prepared by high resolution gel filtration of partial chondroitin ABC lyase digestion of mixed isomer chondroitin sulfate. The main disaccharide repeat in the original chondroitin sulphate is GlcA – GalNAc sulphated at C-6 or C-4 of the GalNAc residue; the CSD disaccharide unit (GlcA,2S – GalNAc,6S) is a minor component comprising approx. 5% of total disaccharides.
**DS oligosaccharides (Iduron)**	These oligosaccharides have been prepared by high resolution gel filtration of partial chondroitin ABC lyase digestion of dermatan sulfate. The main disaccharide in the dermatan sulfate used to prepare these oligosaccharides is IdoA – GalNAc,4S (88% of total disaccharides). The remainder are composed of 7% of disulfated units (IdoA,2S – GalNAc,4S) and 5% non-sulfated disaccharides.
**Heparin 5 kDa (Neoparin Inc.)**	
**Heparin 10.98 kDa (Iduron)**	The 10.98 kDa low molecular weight Heparin is made via partial digestion of heparin with heparinase I, and is purified by size exclusion chromatography.
**Enoxaparin Lovenox® (Sanofi-Aventis)**	The pharmaceutical grade heparin preparation Enoxaparin is prepared by benzylation followed by alkaline depolymerization (Linhardt RJ, Gunay NS. Production and chemical processing of low molecular weight heparins. Semin Thromb Hemost. 1999;25 Suppl 3:5-16)

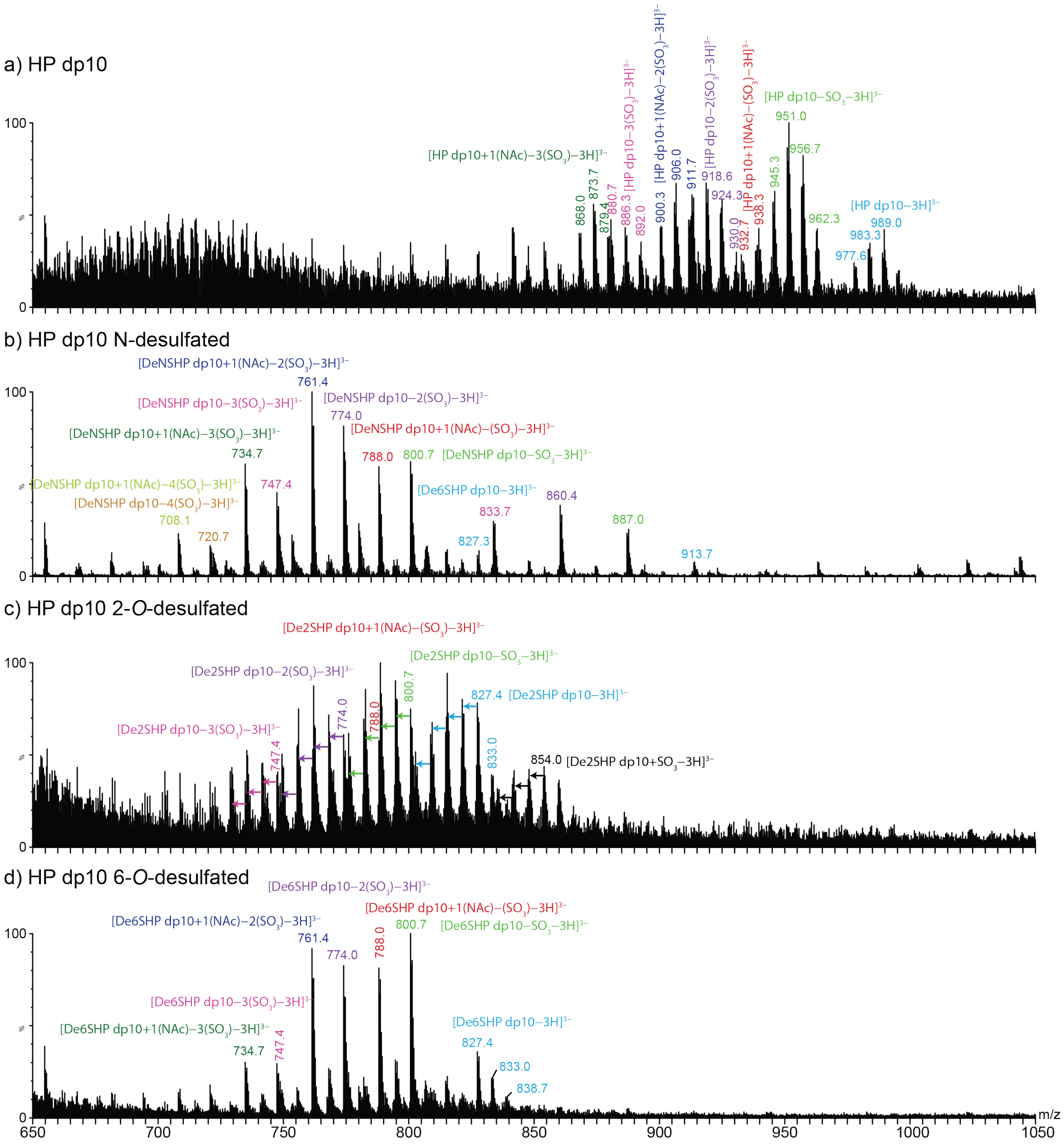

Supplementary Fig. 12 Mass spectra of the regioselectively desulfated heparin dp10 oligosaccharides obtained by hydrophilic liquid chromatography-MS analysis. Mass spectra were summed and zoomed in for the triply charged (3−) ions of the following HP dp10 forms: a) intact, b) *N*-desulfated, c) 2-*O*-desulfated and d) 6-*O*-desulfated. Adduct species are annotated with the same color as the deprotonated species to clarify the spectra. All annotated species were confirmed by exact mass measurement and are presented in **Supplementary Data File 1**. Note that H_2O losses (represented by an arrow) were observed for the HP dp10 2-*O*-desulfated HP dp10 only. Minor sulfate loss occurring during electrospray ionization cannot be ruled out. HILIC-MS experiments were carried out on a Xevo-G2-S Q-TOF mass spectrometer equipped with an electrospray source operating in negative ionization mode. The SeQuant® ZIC®-cHILIC column (3 µm, 100 Å 150 x 2,1 mm) was used with a flow rate of 300 µL/min, and oligosaccharides were eluted using a linear gradient from 23 % of A to 60 % of A in 20 minutes (solvent A: 7.5 mM ammonium formate pH 4.0, solvent B: MeCN).

Supplementary Note 4

Data analysis was performed using Igor Pro 8.04A software (WaveMetrics, OR, USA) with in-house routines. The approach is based on a statistical analysis of current blockades induced by saccharides entering the pore. It involves at least several thousand events. The detection of each current blockade recorded as a time-dependent nanopore current is based on a current-threshold (Th) method. A blockade event is detected when the current magnitude becomes smaller than Th (downward crossing) until it returns to a value greater than Th (upward crossing). It defines the range of points used to compute the characteristic quantities of the blockade, such as the event duration T_t and the mean value I_b (see **Fig. 2**). The threshold value is chosen according to the depth of the event typically $Th = 0.5\ I_0$, where I_0 is the average open-pore current. Before detection of blockades, the current trace is smoothed using a median filter with a smoothing window of 11 points. The average currents I_0 and I_b are measured on the non-filtered trace. The translocation time is defined as the duration between the two crossings of the threshold. Characteristic translocation times were extracted from the long tail of the time distribution by a single-exponential function.

References

1. Kowalczyk, S. W., Wells, D. B., Aksimentiev, A. & Dekker, C. Slowing down DNA translocation through a nanopore in lithium chloride. *Nano Lett.* **12**, 1038–1044 (2012).
2. Fologea, D., Uplinger, J., Thomas, B., McNabb, D. S. & Li, J. Slowing DNA translocation in a solid-state nanopore. *Nano Lett.* **5**, 1734–1737 (2005).
3. Wietelmann, U. & Bauer, R. J. Lithium and Lithium Compounds. in *Ullmann's Encyclopedia of Industrial Chemistry* **21**, 339–366 (Wiley-VCH Verlag GmbH & Co. KGaA, 2012).
