# Supplementary material for: Comprehensive structural assignment of glycosaminoglycan oligo- and polysaccharides by protein nanopore
Source: Nat Commun. 2022 Aug 30;13:5113. doi: 10.1038/s41467-022-32800-4 (PMC9427770; doi:10.1038/s41467-022-32800-4)
Supplement: Supplementary file 2 — Descriptions for additional Supplementary File [file 41467_2022_32800_MOESM2_ESM.pdf]

Supplementary Data 1: Exact mass measurements obtained from HILIC-Q-TOF-MS experiments presented in Supplementary Fig. 12.
